# Terahertz Time-Domain Spectroscopic Characteristics of Typical Metallic Minerals

**DOI:** 10.3390/molecules29030648

**Published:** 2024-01-30

**Authors:** Jingjing Zhang, Haochong Huang, Pengbo Zhao, Luyong Xu, Zhenbo Tan, Jinyuan Zhao, Enhui Yuan, Zhiyuan Zheng, Shanshan Li, Xinyu Li, Kunfeng Qiu

**Affiliations:** 1 School of Science, China University of Geosciences (Beijing), Beijing 100083, China; 17329020494@163.com (J.Z.); ehyuan@cugb.edu.cn (E.Y.); ssli@cugb.edu.cn (S.L.); xyli@cugb.edu.cn (X.L.); 2 School of Earth Science and Resources, China University of Geosciences (Beijing), Beijing 100083, China; zhaopb@cugb.edu.cn (P.Z.); lyxu@cugb.edu.cn (L.X.); zbtan@cugb.edu.cn (Z.T.); jyzhao@cugb.edu.cn (J.Z.); 3 Frontiers Science Center for Deep-Time Digital Earth, China University of Geosciences (Beijing), Beijing 100083, China

**Keywords:** metallic minerals, terahertz time-domain spectroscopy, spectral characterization, lithology, quantitative analysis

## Abstract

Accurate identification and understanding of various metallic minerals are crucial for deciphering geological formations, structures, and ages. Giving their pivotal role as essential natural resources, a microscopic exploration of metallic minerals becomes imperative. Traditional analytical methods, while helpful, exhibit certain limitations. However, terahertz time-domain spectroscopy, distinguished by its high signal-to-noise ratio, expansive frequency band, and low incident wave energy, is a promising complement to conventional techniques in characterizing metallic minerals. This study employs terahertz time-domain spectroscopy to examine samples of Stibnite, Sphalerite, Galena, and Pyrite originating from diverse geological conditions. The vibrations of molecules within these metallic minerals induce discernible changes in the terahertz spectra. Our findings untiate the extensive potential of terahertz time-domain spectroscopy in the characterization of metallic minerals, affirming its considerable practical value in mineral resource exploration.

## 1. Introduction

In 2023, China’s Ministry of Natural Resources presented a solid promotion of a new round of strategic actions to find mineral breakthroughs with substantial initiatives. This proposal highlights the significance of metallic minerals as crucial strategic resources for the country’s scientific and technological progress and economic growth, emphasizing their inseparable connection with China’s economic development and the populace’s standard of living [1,2,3,4]. For example, they play a vital role in various sectors, including the automobile industry, the electronic field, and medical devices. Furthermore, analyzing metallic minerals allows determining an area’s tectonics, structure, and age. Against this backdrop, the analysis and detection of metallic minerals emerge as crucial components in understanding and harnessing these valuable resources [5,6].

Various chemical–physical methods apply to the spectral characterization of rocks and metallic minerals, such as X-ray analysis [7], thermogravimetric analysis [8,9], scanning electron microscope [10], Raman spectroscopy [11], mass spectrometry [12], etc. [13,14] These methods primarily serve as qualitative or semi-quantitative research tools. They utilize waves of various frequency bands to extract molecular vibration modes of substances, generating different data that encapsulate information about the structural components of matter. However, these traditional methods are characterized by their high costs, time-intensive procedures, potential safety hazards, and limitations in accurate mineral identification. For example, X-ray diffraction poses radiation hazards to the human body and is unsuitable for prolonged experimental operations. Similarly, thermogravimetric analysis does not permit sample recovery and reuse after testing.

Terahertz waves, commonly denoting frequencies ranging from 0.1 to 10 THz, inhabit the far-infrared spectrum, positioning themselves between millimeter waves and infrared rays. This frequency range signifies a transition zone bridging macroscopic and microscopic quantum theory realms within the comprehensive electromagnetic spectrum. Due to the specificity of the terahertz electromagnetic spectrum, its interaction with materials has unique physical properties [15,16,17,18]. As an emerging detection method, the terahertz time-domain spectroscopy (THz-TDS) is a non-destructive measurement device [19,20]. It obtains real-time power of terahertz pulses at a high signal-to-noise ratio (SNR) and combines numerous advantages. It possesses high efficiency and speed, low ionization damage, strong penetration, and distinct fingerprinting characteristics [21,22]. This technique is capable of probing crucial phase information in mineral samples and bridging gaps in the field that traditional methods cannot address [23,24], promoting the development of research on the classification and precise analysis of mineral lithologies. Consequently, it holds promise for qualitative and ration evaluation of different minerals [17,21,23,25]. The “Global Mineral Resource Reserve Assessment Report 2023” shows that the level of protection for resources such as tin, lead, zinc, nickel, cobalt, and copper is relatively low. Therefore, in this paper, Stibnite, Sphalerite, Galena and Pyrite from the mining area of Hunan are selected to be investigated using THz-TDS. The results demonstrate that THz-TDS differentiates and identifies mineral samples with varying concentrations, porosities, and masses [26,27,28]. It also offers methodological and technical support for identifying metallic minerals [29], chemical structures, and ration analysis. Due to the high conductivity of metallic minerals, the free electrons can move freely. When the terahertz wave interacts with them, it leads to electrons’ vibration and acceleration, which in turn causes energy loss. Subsequently, the terahertz wave exhibits pronounced absorption within metallic minerals. The absorption effect becomes particularly prominent when the frequency of the terahertz wave aligns with the resonance frequency of free electrons in these minerals, accentuating the overall absorption phenomenon. Consequently, the intensity and configuration of absorption peaks at specific frequencies in the spectrum vary for each mineral component. After that, the terahertz spectra of metallic minerals are scrutinized using a specialized model, enabling a comprehensive study of factors such as molecular structure, crystal composition, electronic state, and more [30,31]. In summary, THz-TDS emerges as a convenient and non-contact method for detecting and identifying metallic minerals, underscoring its substantial practical value in the exploration of mineral resources.

## 2. Experimental Results and Discussion

### 2.1. Mineral Composition and Microstructural Characteristics

**Figure 1:**
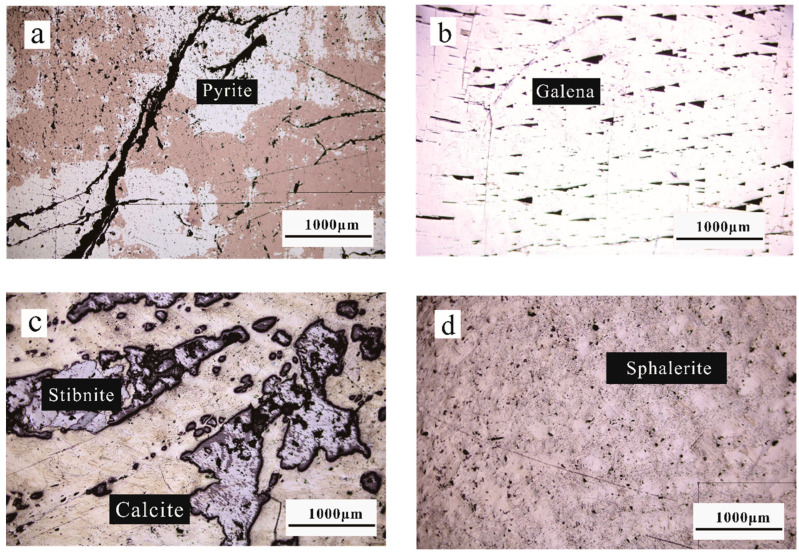
Microscopic characterization of pyrite (**a**), galena (**b**), stibnite (**c**), and sphalerite (**d**) results.

Pyrite, Galena, Stibnite, and Sphalerite are observed under a microscope (Figure 1). Pyrite (Figure 1a) appears yellowish-white under the microscope, with a pockmarked surface, and is a high-hardness mineral because the sulfide is very easy to oxidize; the reddish-brown part is an oxidized film, and it is black because of the low reflectivity of the cleavage. Galena (Figure 1b) is pure white under the microscope, with triangular traps and abrasions on the surface of the mineral, which is a mineral with low hardness. Stibnite (Figure 1c) is gray under the microscope, is an aggregate of elongated bars and grains, with scuff marks seen on the surface, and is a low-hardness mineral. Yellowish-white minerals are nonmetallic calcite, and blackish are Stibnite de-surfaced traps. Sphalerite (Figure 1d) is gray under the microscope, a rare autocrystalline, medium-hardness mineral.

Pyrite, Galena, and Sphalerite exhibit complete extinction under orthogonal light, appearing as equiaxed crystalline homogeneous minerals. In contrast, Stibnite displays strong inhomogeneity. However, it is important to note that the experiment can only provide qualitative observations of minerals, needing more capability for quantitative analysis.

### 2.2. Electron Microprobe Analysis

Analyzing data from electron microprobe experiments yields the elemental contents of the four minerals (refer to Table 1). For each mineral, five distinct observation sites on slides are chosen. The elemental averages are as follows: Pyrite: Fe average = 45.46%, S average = 54.12%; Stibnite: Sb average = 69.3%, S average = 29.24%; Sphalerite: Zn average = 64.66%, S average = 34.2%; Galena: Pb average = 87.71%, S average = 12.18%.

According to Table 2, S/Fe (atomic number) values are 2.039–2.115 in Pyrite. Higher than the standard value of 1.999 for S/Fe (atomic number). Where the Co-to-Ni content ratio is greater than one and less than one, it suggests that Pyrite has undergone hydrothermal and sedimentary genesis; multiple origins and phases of supply may characterize the mineralized material. The elemental content of Sb and S in Stibnite reveals a deviation from the theoretical values (Sb = 71.38%, S = 28.62%), indicating a loss of both Sb and S. The hypothesis posits that these elements integrate into the mineral lattice homogeneously. Pyrite has a small amount of Sb, while Stibnite has traces of Fe, and, probably, the two formed simultaneously. Sphalerite contains relative theoretical values of 67.10% for Zn and 32.90% for S, with loss of Zn with respect to S. Galena has relative theoretical values of 86.60% Pb and 13.40% S, and exhibits Pb-rich characteristics. This suggests that sulfur fugacity changes during mineralization. Some samples contain Zn; crystallizes before Galena, but continues after its crystallization.

**Figure 2:**
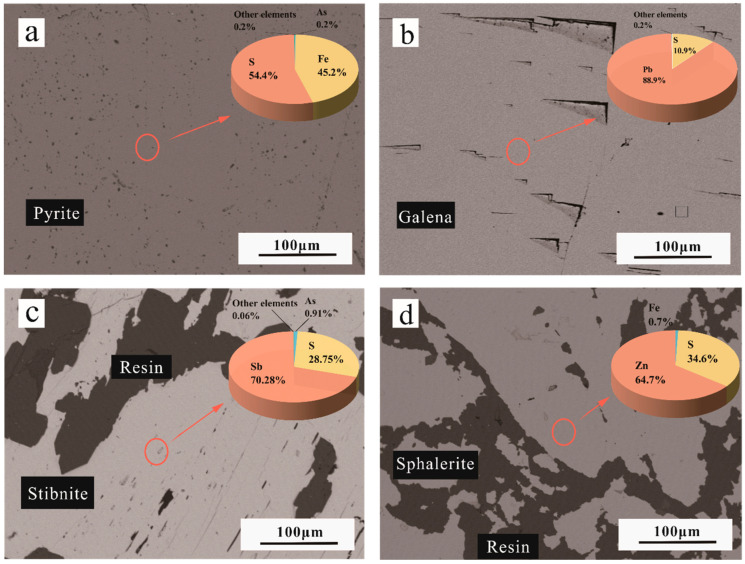
BSE of pyrite (FeS_2_) (**a**), galena (PbS) (**b**), stibnite (Sb_2_S_3_) (**c**) and sphalerite (ZnS) (**d**) results and the corresponding element sectors.

Combining with the backscattered electron (BSE) of the minerals (Figure 2), the purity of the four minerals is high; Pyrite has a regular surface, with star-dotted, scattered resin visible at the edge contacts of the Pyrite crystals; Galena has a nearly right-angled triangular surface bump, with a near-curved distribution of crystal bumps; Stibnite has a large area filled with resin (artificially formed), and the surface is clearly scuffed, suggesting a lower hardness; a large area of resin is present in Sphalerite as well. Analyzing the elemental fan diagrams at the respective points reveals that the primary chemical compositions of Pyrite, Galena, Stibnite, and Sphalerite are FeS_2_, PbS, Sb_2_S_3_, and ZnS. However, it is worth noting that this method proves to be time-consuming and expensive.

### 2.3. THz-TDS

#### 2.3.1. Mineral Spectral Characterization

**Figure 3:**
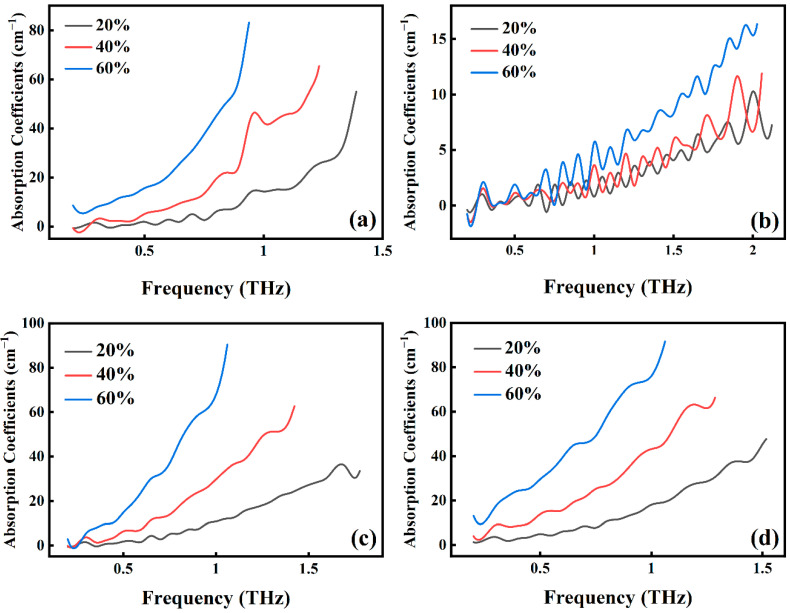
Absorption coefficients for different concentrations of stibnite (**a**), sphalerite (**b**), galena (**c**), and pyrite (**d**).

This study uses THz-TDS to analyze different concentrations of Stibnite, Sphalerite, Galena, and Pyrite The absorption coefficient curves of Stibnite (Figure 3a), Galena (Figure 3c) and Pyrite (Figure 3d) at a 60% concentration exhibit significantly higher values compared to those at a 20% concentration within the 0.2–1.5 THz range. The average absorption coefficient values for Stibnite, Galena, and Pyrite at a 60% concentration are determined to be 25.88, 31.28, and 43.84, respectively, while those at a 20% concentration are 10.49, 13.16, and 16.16, respectively. This observation demonstrates that the absorption coefficients increase with the increase in mineral concentration, aligning with Beer–Lambert law [32].

**Figure 4:**
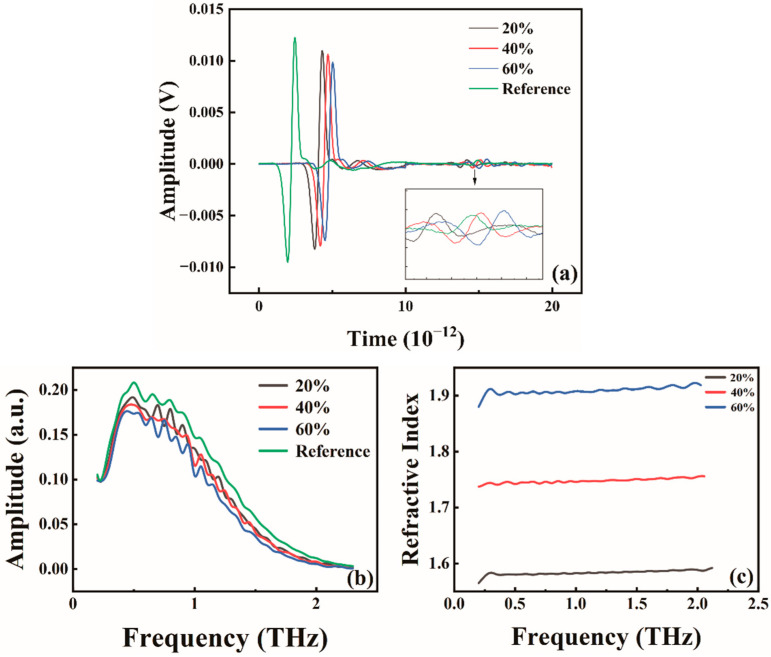
Terahertz time-domain waveforms (**a**) terahertz frequency-domain amplitude (**b**) and refractive index (**c**) of sphalerite samples at different concentrations.

However, the absorption coefficient curves of Sphalerite (Figure 4b) exhibit irregular fluctuations, illustrated in Figure 4a. The time-domain signals of the three samples (20%, 40%, and 60%) contain echo signals, and their unprocessed amplitude curves obtained directly by Fourier transform (Figure 4b) also display irregular fluctuations. Consequently, the absorption coefficient plots (Figure 3b) and the refractive index plots (Figure 4c), obtained through calculation, exhibit more pronounced irregular fluctuations. Moreover, the average values of the absorption coefficients for concentrations of 80%, 60%, and 20% are 5.99, 3.49, and 3.07, respectively, indicating that they still adhere to the principle that increasing concentration enhances the absorption coefficients. However, due to the uncertainty associated with the optical frequency at low and high frequencies, the concentration size for the remaining groups of samples is ultimately determined to be 40%.

**Figure 5:**
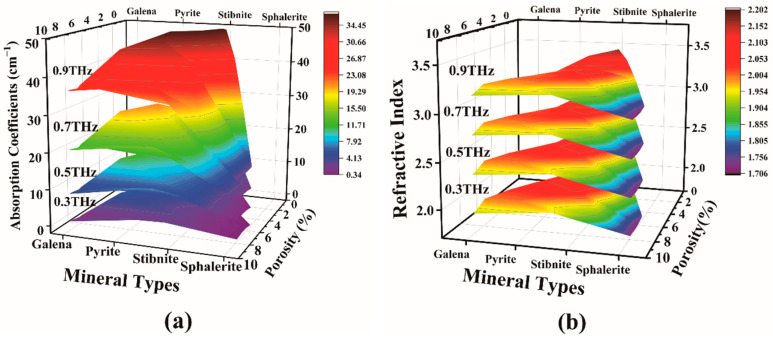
The dependence of absorption coefficients (**a**) and refraction index (**b**) on sample type and porosity at 0.3, 0.5, 0.7, and 0.9 THz.

By analyzing the slow slope take, 0.3, 0.5, 0.7, and 0.9 THz are chosen as the absorption coefficient values at the projection point. Figure 5a comprises absorption coefficients, sample porosity, and species. At a frequency of 0.3 THz, the absorption coefficient of Galena, Pyrite, Stibnite, and Sphalerite remain in the range of 0.99–2.49, 4.04–6.43, 0.88–1.35, 0–0.36, respectively, as the porosity increases. Similarly, conducted within a limited range, the analysis focused on absorption coefficient values for minerals at 0.5, 0.7 and 0.9 THz. As depicted in Figure 5, the absorption coefficient values of minerals exhibit a consistent pattern, maintaining relative constancy across the spectrum from low to high porosity. As the frequency rises, the mapping surface colors of Galena and Pyrite shift from blue to red, and Stibnite transitions from blue to green. This change signifies a gradual increase in their absorption coefficient values. In the case of Sphalerite, the color remains predominantly bluish-purple, with a minor increment in absorption coefficients.

The overall adsorption rate demonstrates improvement with an increase in sample frequency. Negative absorption coefficients in Sphalerite can be attributed to the introduction of noise during its measurement using THz-TDS. Additionally, the absorption rate of Stibnite is the highest, while Sphalerite is significantly lower than the other three minerals. When the porosity ranges from 5–8%, the maximum value of Sphalerite absorption coefficients does not exceed 5 cm^−1^, as analyzed in Figure 4. Sphalerite’s mineral properties, composition, and content contribute to this behavior. Consequently, terahertz creates favorable conditions for qualitative and quantitative detection of substances.

In Figure 5b, the relationship between the refractive index and porosity of the samples reveals that cassiterite occupies the uppermost position while Sphalerite settles at the bottom. Furthermore, the refractive index gradually increases from the higher side towards samples with lower porosity. Additionally, it is worth noting that the refractive index of minerals is directly proportional to frequency. To illustrate this principle and investigate the connection between frequency and absorption coefficients while also minimizing measurement systematic errors, we analyzed the average absorption coefficients for samples with porosities of 1.95% for Stibnite, 7.8% for Sphalerite, 8.9% for Galena, and 4.2% for Pyrite.

**Figure 6:**
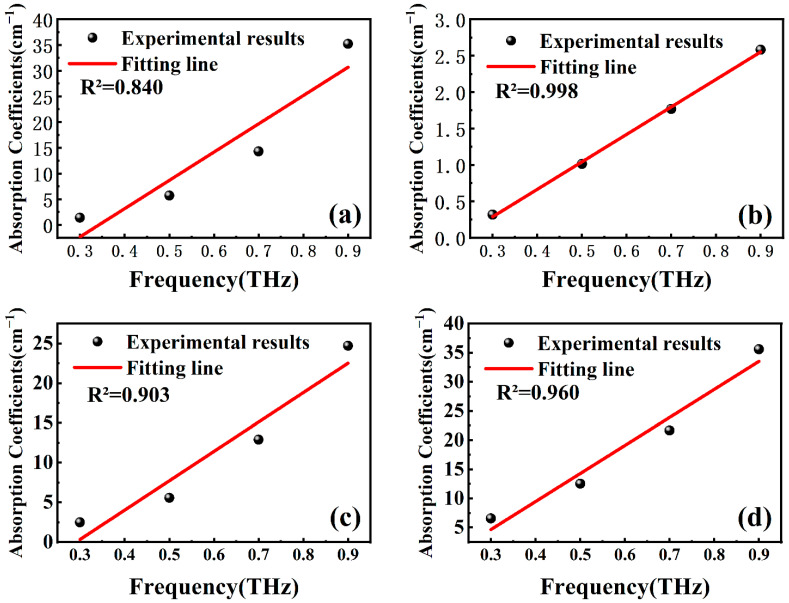
Frequency-absorption coefficient fits for 1.95% porosity stibnite (**a**), 7.8% sphalerite (**b**), 8.9% galena (**c**), and 4.2% pyrite (**d**).

Figure 6 shows the results of the linear fit to the frequency-absorption coefficients. The linear relationship between the absorption coefficients and frequency is robust with an R^2^ value of 0.998 at a porosity of 7.8%. Similarly, for Stibnite with a porosity of 1.95%, Galena with 8.9%, and Pyrite with 4.2%, the frequencies and absorption coefficients show a proportional relationship, with R^2^ values of 0.84, 0.903, and 0.960, respectively.

**Figure 7:**
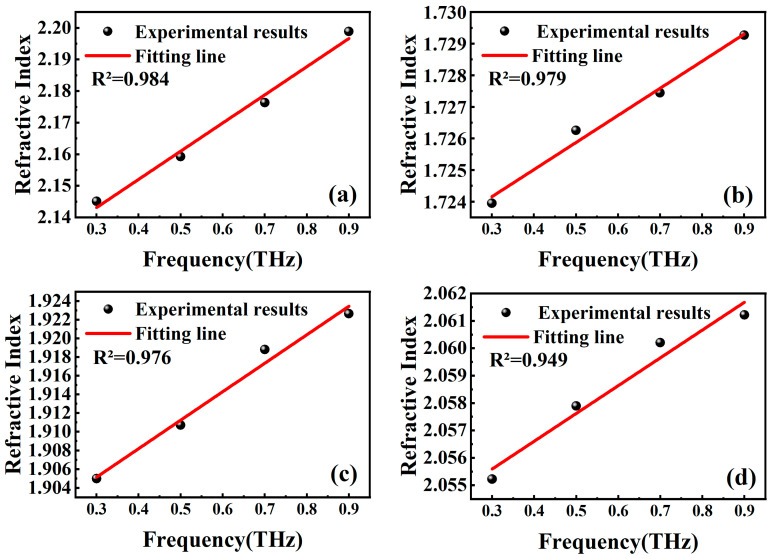
Frequency-refractive index fits for 1.95% porosity stibnite (**a**), 7.8% sphalerite (**b**), 8.9% galena (**c**), and 4.2% pyrite (**d**).

Upon fitting the refractive index versus frequency once again, it is observed from the results depicted in Figure 7 that the correlation coefficients for Stibnite, Sphalerite, Galena, and Pyrite all exceed 0.945. This value indicates a strong linear relationship between the refractive index and the frequency of the samples.

#### 2.3.2. Analysis Using THz-TDS

**Figure 8:**
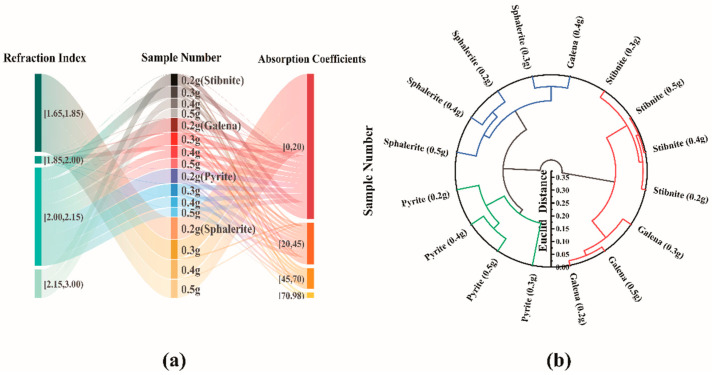
Sankey plots of absorption coefficients and refractive indices for different masses of stibnite, galena, pyrite, and sphalerite (**a**) and circular clustering plots based on refractive index spectra in the 0.1–1.5 THz band (**b**).

The absorption coefficients of the 16 different samples in Figure 8a at 0–2.5 THz frequencies exhibit varying degrees of variation. These variations suggest that the mineral samples’ unique elemental compositions and structures significantly manifest in the terahertz band. In contrast, Sphalerite exhibits the lowest absorption coefficients across the entire band, with 96.67% of the Sphalerite absorption coefficients falling between 0 and 20 cm^−1^ and 3.33% between 20 and 45 cm^−1^. The other three minerals, on the other hand, demonstrate relatively concentrated absorption coefficients, accounting for 19.38%, 22.42%, and 21.46% of the total data volume, respectively. These proportions remain consistent across various intervals, posing challenges in their rapid differentiation.

In the terahertz band, the refractive index of the samples exhibits variations. Specifically, the refractive index of Sphalerite remains constant between 1.62 and 1.78 across the entire band. Additionally, Pyrite shows a refractive index value of 67.94%, primarily within the interval of 2.15–3.0. On the other hand, the refractive index values of Pyrite and Galena predominantly fall within the range of 2.00–2.15, accounting for 86.82% and 96.77%, respectively. Consequently, discerning their mineral refractive index values becomes challenging due to their relatively concentrated nature. In contrast, the refractive index of Sphalerite and Stibnite stands out as the most distinctive among the other minerals. Hence, in the rapid identification of minerals, it is advisable first to calibrate the refractive index of Sphalerite and Stibnite using terahertz technology.

Classification studies using terahertz spectral data have employed cluster analysis to qualitatively analyze the similarities between 16 mineral specimens and the effects of different compositions on the terahertz parameters [32,33,34,35]. The Euclidean distance clustering algorithm used in this paper is an unsupervised learning method; the idea is to categorize sample points that are closer in the sample space into classes. The Euclidean distance can reflect the absolute differences in individual numerical magnitudes and effectively capture differences in the numerical magnitude of the dimensions. This approach minimizes intra-class differences while maximizing inter-class differences [36]. This process entails five step-by-step clustering procedures, resulting in a pair of clustering tree diagrams that visually illustrate the similarities and dissimilarities among the 16 mineral samples (refer to Figure 8b).

The samples pertain to three categories. The first category contains three samples of Galena (0.2 g, 0.3 g, 0.5 g) and four samples of Stibnite (0.2 g, 0.3 g, 0.4 g, 0.5 g). The Euclidean distance of 0.006 between the 0.4 g and 0.5 g Stibnite specimens in this category indicates their highest similarity. The second category consists of four Sphalerite (0.2 g, 0.3 g, 0.4 g, 0.5 g) and one Galena (0.4 g). In this category, the smallest Euclidean distance of 0.046 is observed between the 0.4 g Galena model and 0.3 g Sphalerite sample, suggesting their highest similarity. The third category includes four Pyrite samples (0.2 g, 0.3 g, 0.4 g, and 0.5 g). The minimum Euclidean distance of 0.057 is found between the 0.4 g and 0.5 g Pyrite samples. Overall, the cluster analysis results align with the mineral lithology, except for the slight difference observed in the 0.4 g Galena sample. Galena is in the same group as Stibnite due to its chemical composition and crystal structure, which contains elements like lead (Pb) or antimony (Sb). These shared elements result in similarities in specific properties. The resemblance in crystal structures or lattice types between the two minerals may lead to similarities in their response to electromagnetic waves in the terahertz band, including the refractive index Therefore, the cluster analysis results generally support the lithology of the specimens, except for the slight difference observed in the Galena 0.4 g sample.

**Figure 9:**
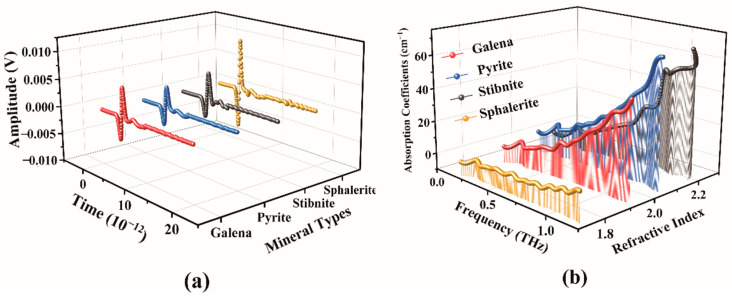
Time-domain amplitude plots (**a**) and absorption coefficients-refractive index plots (**b**) for galena, pyrite, stibnite, and sphalerite.

Four mineral samples, each with a concentration of 40% and pressed at 10 MPa, have been selected for further study. Based on the THz-TDS depicted in Figure 9a, it is evident that the four minerals exhibit unique terahertz spectral characteristics. Furthermore, the amplitudes of these minerals differ significantly within the effective period, indicating variations in their absorption and transmission properties under terahertz waves. Distinct composition and content among the samples contribute to the observed signal delay, influencing the refractive index and terahertz waves’ absorption and propagation. Notably, the waveforms of Sphalerite demonstrate substantial amplitude variations, underscoring its pronounced absorption and scattering capabilities concerning terahertz waves. Conversely, Pyrite, Galena, and Stibnite waveforms exhibit comparably smaller amplitude variations than Sphalerite. Hence, these minerals possess discernible mineral compositions.

After performing prior calculations, calculate the optical constants of these four minerals to help distinguish them. Following the organization of data and integration of images, absorption coefficients and refractive index plots for the four minerals are derived. The figure shows that the four minerals’ absorption coefficients gradually increase with an increase in terahertz wave frequency. This phenomenon is attributed to the shorter wavelength resulting from the higher frequency of terahertz waves, causing the minerals to absorb more energy. Notably, Sphalerite shows a considerably smaller variation than the other three minerals, with only a slight increase in absorption coefficients. This smaller variation in Sphalerite occurs due to Sphalerite’s high transmittance, resulting in a weaker absorption capacity for terahertz waves. Conversely, the absorption coefficients of the other three minerals exhibit significant changes with increased terahertz wave frequency. Specifically, Sphalerite, Galena, and Pyrite display distinct absorption curves that overlap at 0.2–0.9 THz and 0.9–1.4 THz. The identical composition and content in the samples lead to this overlap.

The variation in absorption coefficients allows for a relatively clear distinction between Sphalerite and the other three minerals. When analyzing the mineral refractive index, there are no apparent significant changes as the terahertz wave frequency varies. However, when comparing the four minerals, there are noticeable differences in their respective refractive indexes. Sphalerite has the lowest refractive index, followed by Galena, Pyrite, and Stibnite in descending order. Sphalerite, composed of zinc sulfide (ZnS), exhibits a low refractive index in the terahertz range. This low refractive index suggests the presence of electron jumps within the molecular structure of Sphalerite, leading to absorption and scattering at terahertz frequencies, consequently resulting in a relatively low refractive index. Conversely, Sphalerite possesses the highest refractive index. The hypothesis is that the molecular structure of Sphalerite chemical composition may contain free-moving electrons, contributing to the stronger oscillation at the corresponding terahertz wave frequency and yielding a higher refractive index for the mineral.

The above analysis shows that the structure and composition of various minerals differ significantly, exhibiting unique terahertz spectral characteristics. These characteristics serve as symbols for the physical properties and disparities among minerals, highlighting the potential of THz-TDS as a novel technology for qualitative analysis of metallic minerals.

## 3. Methods

### 3.1. Microscopy

Antimony Sphalerite and Pyrite from tin mines in Leng Shui Jiang City, Hunan Province, and Galena and Sphalerite from Lin Wu deposits in Hunan Province were selected for the experiments, and cut and polished into light sheets of 30 mm × 50 mm in size and 75 μm in thickness. Then, an Olympus microscope scrutinized the sheets at 50 times magnification. The preliminary mineral composition of the sample was determined and its structure was observed [37,38]. The preparation process of the light sheet was as follows:

(1) Cut: partition the ore specimen for observation into pieces of a specific size on a slicing machine.
(2) Ground: after washing the cut pieces of ore with water, the specimen and the slide were ground on a grinder so that the cut surface of the sample became a smooth plane, after which it was glued.
(3) Sliced: after the gel had hardened, the specimen was placed in a thin-section cutter, which cut and ground it to a 100–150 μm thickness.
(4) Fine ground: the rock specimen was ground on a grinder to a thickness of 75 μm.
(5) Polished: put the finely ground ore on the canvas grinding disk and the tweed grinding disk, and add the water-tuned MgO for polishing, until the surface is as smooth as a mirror.

### 3.2. Electron Microprobe Analysis

In this experiment, a carbon film was sprayed on the surface of a light sheet (the one made in Section 3.1) and five different points were selected for quantitative and qualitative analysis of trace elements in the minerals using an electron microprobe analysis (EMPA) model EMPA-1600 Shimadzu, Japan [39,40]. The operating conditions included an accelerating voltage of 15 kV, a beam current of 10 nA, and an electron beam diameter of 5 μm.

### 3.3. THz-TDS

A transmission terahertz time-domain spectroscopy system, comprising a femtosecond laser, a photoconductive antenna, a time-delay control system, and other corresponding equipment, was utilized in this study. The femtosecond laser comprises a titanium sapphire laser with a center wavelength of 800 nm, a pulse width of 100 fs, and a repetition frequency of 80 MHz. The laser featured a spectral range of 0.1–3.5 THz and an average energy output of 500 mW. This system facilitated the detection of ample spectral information, enabling the analysis of materials’ physical and chemical properties [41]. The fundamental principle involved the terahertz electric field altering the detected light’s polarization state, modulating the detector crystal’s refractive index. The system, in turn, converted the light into an electrical signal, providing information about the electric field strength. A subsequent Fourier transform was applied to obtain amplitude and phase information in the broadband frequency domain, contingent upon meeting the requirements for the phase data. Entering the corresponding formulas yielded the absorption coefficients (*α*(*w*)) and refractive index (n(*w*)) of the samples [42,43].

$$n\left(w\right)=\frac{\varphi \left(w\right)c}{wd}+1\;\alpha \left(w\right)=\frac{2}{d}\ln\left\{\frac{\begin{array}{c} 4n\left(w\right) \end{array}}{A\left(w\right){\left[n\left(w\right)+1\right]}^{2}}\right\}$$

Here, *A*(*w*) is the amplitude ratio of the sample signal to the reference signal, *φ*(*w*) is the phase difference between the sample and the reference signal, *c* is the speed of light, and *d* was the total penetration thickness of the terahertz wave. THz-TDS had high environmental requirements and was placed in an ultra-clean room, maintaining a room temperature of 22 degrees Celsius.

#### Sample Preparation

Initially, these minerals (referred to in Section 3.1) were ground into a powder below 200 mesh, followed by sample preparation using the pressing method. The specific steps for sample preparation were as follows:

(1) Ground: the bulk sample was placed into the appropriate grinding device and periodically operated to achieve a particle size range within 200 mesh or below.
(2) Sieved: the powdered samples were filtered through a sieve to ensure they were below 200 mesh.
(3) Dried: before sizing, the mineral powders undergo drying in an oven to eliminate any adverse effects of moisture on the characterization results.
(4) Prepared: in Group I, the powder of the four metal samples was mixed with Polytetrafluoroethylene (PTFE) powder in different ratios of 2:8, 4:6, and 6:4 to control the concentration of the metal minerals and porosity. The weighed samples had a mass of 0.3 g and were prepared using a tablet press pressure of 4 MPa, resulting in a specimen thickness of approximately 0.8 mm. Moving on to Group II, the powder of the four metal samples was mixed with PTFE powder in a 4:6 ratio. Sample preparation involved applying press pressures of 4 MPa, 7 MPa, 10 MPa, 13 MPa, 16 MPa, and 19 MPa, while the mass of the weighed samples remained at 0.3 g. Lastly, in Group III, the powder of the four metal samples was mixed with PTFE powder in a 4:6 ratio, and a press pressure of 19 MPa was applied for the preparation of Stibnite, Sphalerite, Galena, and Pyrite samples, all had the same particle size. The mass of the samples varied at 0.2 g, 0.3 g, 0.4 g, and 0.5 g, and Table 3 presented their corresponding thicknesses.

## 4. Conclusions

This paper describes the analysis of four metallic mineral samples using THz-TDS in combination with conventional means. The simple qualitative observations of the composition and structure of the samples under a microscope did not allow for their quantitative analysis. In the EMPA experiment, the analysis of the elements’ content determined the minerals’ composition and the genesis of their deposits. However, the method is costly and it is time-consuming to prepare samples. Analyzed using THz-TDS, the terahertz spectra of the samples are obtained through the interaction of terahertz light with the molecular vibrations in the metallic minerals. The calculation of absorption coefficients and refractive index for terahertz waves under varying conditions (porosity, concentration, and mass) is conducted, followed by a qualitative analysis of the specimens. The findings demonstrate that THz-TDS is an effective method for analyzing mineral properties. By employing absorption coefficients and refractive index plots, specific to the four minerals, in conjunction with the cluster analysis method, the results highlight discernible differences in terahertz optical parameter spectra for minerals with distinct compositional contents. Thus, THz-TDS can capture subtle changes in molecular type and structure, enabling the modeling of mineral material composition and the content of lead, zinc, and other metal elements. This study established the effectiveness of THz-TDS for the identification and quantitative analysis of metallic minerals, offering a new technology for mineral resource exploration. In conclusion, THz-TDS provides a convenient, non-contact method for detecting and identifying metallic minerals; it is of great value in the search for mineral resources of great economic value and the analysis and processing of mineral resources, determining the geological age and type of minerals, etc. This novel technique for analyzing deposit patterns and mineralization mechanisms introduces new analytical approaches to geological exploration.

## Figures and Tables

**Table 1:** Quantitative electron microprobe results for pyrite, stibnite, sphalerite, and galena at five different measuring points (wt %).

**Pyrite**											
**Point**	**As**	**Fe**	**S**	**Ni**	**Co**	**Pb**	**Cu**	**Ag**	**Zn**	**Sb**	**Total**
1	0.19	45.48	54.36	0.00	0.06	0.00	0.05	0.00	0.06	0.01	100.22
2	0.22	45.11	54.35	0.02	0.09	0.00	0.04	0.03	0.00	0.00	99.85
3	0.28	45.00	54.41	0.03	0.18	0.00	0.03	0.03	0.00	0.01	99.96
4	0.24	45.41	53.30	0.00	0.08	0.00	0.01	0.02	0.02	0.00	99.09
5	0.25	46.30	54.19	0.00	0.08	0.00	0.05	0.00	0.02	0.00	100.89
**Stibnite**											
**Point**	**As**	**Fe**	**S**	**Ni**	**Co**	**Pb**	**Cu**	**Ag**	**Zn**	**Sb**	**Total**
1	0.88	0.00	28.42	0.05	0.03	0.00	0.00	0.02	0.00	69.58	98.97
2	0.90	0.01	28.42	0.01	0.00	0.00	0.02	0.02	0.00	69.47	98.85
3	0.01	0.00	0.01	0.03	0.00	0.00	0.02	0.00	0.00	0.04	0.11
4	0.00	0.00	0.01	0.04	0.00	0.00	0.00	0.00	0.00	0.06	0.10
5	0.72	0.00	30.90	0.05	0.02	0.00	0.00	0.00	0.00	68.86	100.56
**Sphalerite**											
**Point**	**As**	**Fe**	**S**	**Ni**	**Co**	**Pb**	**Cu**	**Ag**	**Zn**	**Sb**	**Total**
1	0.00	0.66	34.26	0.00	0.02	0.00	0.00	0.00	64.68	0.01	99.62
2	0.00	0.70	34.72	0.00	0.04	0.00	0.00	0.00	65.01	0.00	100.47
3	0.00	0.66	33.63	0.00	0.00	0.00	0.00	0.05	64.29	0.03	98.64
4	0.01	0.01	0.00	0.00	0.01	0.00	0.00	0.00	0.33	0.00	0.35
5	0.00	0.00	0.01	0.02	0.02	0.00	0.00	0.00	0.78	0.00	0.83
**Galena**											
**Point**	**As**	**Fe**	**S**	**Ni**	**Co**	**Pb**	**Cu**	**Ag**	**Zn**	**Sb**	**Total**
1	0.00	0.02	13.18	0.07	0.00	87.32	0.00	0.00	0.04	0.02	100.64
2	0.00	0.00	10.82	0.02	0.00	88.06	0.10	0.00	0.08	0.00	99.07
3	0.00	0.03	10.82	0.00	0.00	88.01	0.03	0.00	0.00	0.01	98.89
4	0.00	0.00	13.01	0.00	0.07	87.61	0.03	0.00	0.00	0.00	100.72
5	0.00	0.00	13.08	0.06	0.00	87.58	0.00	0.00	0.00	0.00	100.71

**Table 2:** Molar percentage of pyrite, stibnite, sphalerite and galena (wt %).

	**Pyrite**					**Stibnite**		
**Point**	**1**	**2**	**3**	**4**	**5**	**1**	**2**	**5**
Fe (%)	32.40	32.22	32.14	32.80	32.86			
Sb (%)						39.15	39.11	36.93
S (%)	67.59	67.77	67.85	67.19	67.13	60.84	60.88	63.06
	**Galena**					**Sphalerite**		
**Point**	**1**	**2**	**3**	**4**	**5**	**1**	**2**	**3**
Pb (%)	50.58	55.69	55.67	50.98	50.84			
Zn (%)						48.02	47.82	48.34
S (%)	51.97	52.17	51.65	49.41	44.3	44.32	49.01	49.15

**Table 3:** The porosity and thickness of samples prepared by four metallic minerals under different pressures.

**Sample Name**	**4 MPa**		**7 MPa**		**10 MPa**	
	**Porosity** **(%)**	**Thickness** **(mm)**	**Porosity** **(%)**	**Thickness** **(mm)**	**Porosity** **(%)**	**Thickness** **(mm)**
**Galena**	9.16	0.81	8.98	0.81	8.9	0.81
**Pyrite**	6.43	0.87	4.82	0.83	4.42	0.83
**Stibnite**	5.12	0.88	3.36	0.86	2.45	0.85
**Sphalerite**	7.8	0.91	7.62	0.91	7.21	0.91
**Sample Name**	**13 MPa**		**16 MPa**		**19 MPa**	
	**Porosity** **(%)**	**Thickness** **(mm)**	**Porosity** **(%)**	**Thickness** **(mm)**	**Porosity** **(%)**	**Thickness** **(mm)**
**Galena**	8.15	0.79	8.02	0.79	7.75	0.79
**Pyrite**	4.16	0.83	4.13	0.83	4.8	0.82
**Stibnite**	1.95	0.84	1.13	0.83	0.8	0.82
**Sphalerite**	6.8	0.9	6.07	0.88	5.22	0.88

## Data Availability

Data are contained within the article.

## References

[1] Dubiński J. (2013). Sustainable Development of Mining Mineral Resources. J. Sustain. Min..

[2] Aryee B.N.A. (2001). Ghana’s Mining Sector: Its Contribution to the National Economy. Resour. Policy.

[3] Zhou B., Li Z., Chen C. (2017). Global Potential of Rare Earth Resources and Rare Earth Demand from Clean Technologies. Minerals.

[4] Zhou L. (2023). Towards Sustainability in Mineral Resources. Ore Geol. Rev..

[5] Wuana R.A., Okieimen F.E. (2011). Heavy Metals in Contaminated Soils: A Review of Sources, Chemistry, Risks and Best Available Strategies for Remediation. ISRN Ecol..

[6] Liu Y., Wu A., Wang J., Taghizadeh-Hesary F., Dong X. (2024). Green Growth in the Global South: How Does Metallic Minerals Affect GTFP Enhancement?. Resour. Policy.

[7] Sinforiano S.-R., Antonio D.-C., José Vicente G.-A., Juan P.-V. (2008). Analytical and Mineralogical Studies of Ore and Impurities from a Chromite Mineral Using X-Ray Analysis, Electrochemical and Microscopy Techniques. Talanta.

[8] Marshall C.P., Kamali S., Wilson M., Guerbois J.P., Hartung-Kagi B., Hart G. (2002). Potential of Thermogravimetric Analysis Coupled with Mass Spectrometry for the Evaluation of Kerogen in Source Rocks. Chem. Geol..

[9] Deboucha W., Leklou N., Khelidj A., Oudjit M.N. (2017). Hydration Development of Mineral Additives Blended Cement Using Thermogravimetric Analysis (TGA): Methodology of Calculating the Degree of Hydration. Constr. Build. Mater..

[10] Karato S.I. (1987). Scanning Electron Microscope Observation of Dislocations in Olivine. Phys. Chem. Miner..

[11] Kim H.I., Cho H.G., Lee S., Koo H.J., Hong J.K., Jin Y.K. (2023). Spatial Distribution of Manganese Oxide Minerals in the Natural Ferromanganese Nodule of the Arctic Sea: A View from Raman Spectroscopy. Chem. Geol..

[12] Kemp S.J., Lewis A.L., Rushton J.C. (2022). Detection and Quantification of Low Levels of Carbonate Mineral Species Using Thermogravimetric-Mass Spectrometry to Validate CO_2_ Drawdown via Enhanced Rock Weathering. Appl. Geochem..

[13] Huang H., Yuan E.-H., Zhang D., Sun D., Yang M., Zheng Z., Zhang Z., Gao L., Panezai S., Qiu K. (2023). Free Field of View Infrared Digital Holography for Mineral Crystallization. Cryst. Growth Des..

[14] Siidra O.I., Nekrasova D.O., Depmeier W., Chukanov N.V., Zaitsev A.N., Turner R.W. (2018). Hydrocerussite-Related Minerals and Materials: Structural Principles, Chemical Variations and Infrared Spectroscopy. Acta Crystallogr. Sect. B Struct. Sci. Cryst. Eng. Mater..

[15] Baxter J.B., Guglietta G.W. (2011). Terahertz Spectroscopy. Anal. Chem..

[16] Manjappa M., Singh R. (2020). Materials for Terahertz Optical Science and Technology. Adv. Opt. Mater..

[17] Heshmat B., Andrews G.M., Naranjo-Montoya O.A., Castro-Camus E., Ciceri D., Sanchez A.R., Allanore A., Kmetz A.A., Eichmann S.L., Poitzsch M.E. (2017). Terahertz Scattering and Water Absorption for Porosimetry. Opt. Express.

[18] Qu F., Nie P., Liang L., Cai C., He Y. (2018). Review of Theoretical Methods and Research Aspects for Detecting Leaf Water Content Using Terahertz Spectroscopy and Imaging. Int. J. Agric. Biol. Eng..

[19] Siegel P.H. (2002). Terahertz Technology. IEEE Trans. Microw. Theory Tech..

[20] Zhang N., Lim S.J., Toh J.M., Wei Y.F., Rusli, Ke L. (2022). Investigation of Spoilage in Salmon by Electrochemical Impedance Spectroscopy and Time-Domain Terahertz Spectroscopy. ChemPhysMater.

[21] Yin X., Feng M., Jiang Y., Chen T. (2021). Quantitative Analysis of the 2-Mercaptobenzothiazole Based on Terahertz Time-Domain Spectroscopy and an Improved Support Vector Regression. Infrared Phy. Technol..

[22] Ge L.N., Zhan H., Leng W.X., Zhao K., Xiao L. (2015). Optical Characterization of the Principal Hydrocarbon Components in Natural Gas Using Terahertz Spectroscopy. Energy Fuels.

[23] Yang M., Zhang S.-Q., Huang H., Ma Y., Hao S., Zhang Z., Zheng Z. (2022). Insights into a Mineral Resource Chlorite Mica Carbonate Schist by Terahertz Spectroscopy Technology. Energies.

[24] Li S., Qiu K., Hernández-Uribe D., Gao Y., Santosh M., Huang H., Zheng Z., Zhang Z., Gao S. (2023). Water Recycling in the Deep Earth: Insights from Integrated μ-XRF, THz-TDS Spectroscopy, TG, and DCS of High-Pressure Granulite. J. Geophys. Res. Solid Earth.

[25] Zhan H., Wu S., Bao R., Ge L. (2015). Qualitative Identification of Crude Oils from Different Oil Fields Using Terahertz Time-Domain Spectroscopy. Fuel.

[26] Kiritharan S., Lucas S., Degl’Innocenti R., Xia H., Dawson R., Lin H. (2024). Porosity Characterisation of Solid-State Battery Electrolyte with Terahertz Time-Domain Spectroscopy. J. Power Sources.

[27] Bretz L., Niehues G., Funkner S., Bründermann E., Müller A.-S., Lanza G. (2023). In-Line Measurement of Fiber Mass Fraction Using Terahertz Spectroscopy for a Function-Oriented Quality Assurance of Glass Fiber Sheet Molding Compound. Measurement.

[28] Cheng H., Huang H., Yang M., Yang M., Yan H., Panezai S., Zheng Z., Zhang Z., Zhang Z.L. (2022). Characterization of the Remediation of Chromium Ion Contamination with Bentonite by Terahertz Time-Domain Spectroscopy. Sci. Rep..

[29] Han D., Jeong H., Song Y., Ahn J.S., Ahn J. (2015). Lattice Vibrations of Natural Seraphinite Gemstone Probed by Terahertz Time-Domain Spectroscopy. IEEE Trans. Terahertz Sci. Technol..

[30] Bao R., Wu S., Zhao K., Zheng L., Xu C.-H. (2013). Applying Terahertz Time-Domain Spectroscopy to Probe the Evolution of Kerogen in Close Pyrolysis Systems. Sci. China Phys. Mech. Astron..

[31] Ohkoshi S., Yoshikiyo M., Namai A., Nakagawa K., Chiba K., Fujiwara R., Tokoro H. (2017). Cesium Ion Detection by Terahertz Light. Sci. Rep..

[32] Casasanta G., Falcini F., Garra R. (2022). Beer–Lambert Law in Photochemistry: A New Approach. J. Photochem. Photobiol. A Chem..

[33] Stahl D., Sallis H. (2012). Model-Based Cluster Analysis. Wiley Interdiscip. Rev. Comput. Stat..

[34] Calinski T., Harabasz J. (1974). A Dendrite Method for Cluster Analysis. Commun. Stat.-Theory Methods.

[35] Fraley C. (1998). How Many Clusters? Which Clustering Method? Answers via Model-Based Cluster Analysis. Comput. J..

[36] Han P., Wang W., Shi Q., Yue J. (2021). A Combined Online-Learning Model with K-Means Clustering and GRU Neural Networks for Trajectory Prediction. Ad Hoc Netw..

[37] Han Z., Zhang B., Wu H., Liu H., Qiao Y., Zhang S. (2020). Microscopic Characterisation of Metallic Nanoparticles in Ore Rocks, Fault Gouge and Geogas from the Shanggong Gold Deposit, China. J. Geochem. Explor..

[38] Wei Y., Li H., Yamada N., Sato A., Ninomiya Y., Honma K., Tanosaki T. (2013). A Microscopic Study of the Precipitation of Metallic Iron in Slag from Iron-Rich Coal during High Temperature Gasification. Fuel.

[39] Keil K., Fredriksson K. (1963). Electron Microprobe Analysis of Some Rare Minerals in the Norton County Achondrite. Geochim. Cosmochim. Acta.

[40] Velde B. (1984). Electron Microprobe Analysis of Clay Minerals. Clay Miner..

[41] Ferguson B., Zhang X.-C. (2002). Materials for Terahertz Science and Technology. Nat. Mater..

[42] Duvillaret L., Garet F., Coutaz J.-L. (1996). A Reliable Method for Extraction of Material Parameters in Terahertz Time-Domain Spectroscopy. IEEE J. Sel. Top. Quantum Electron..

[43] Jiang X., Xu Y., Hang H., Xie W. (2023). Nondestructive Testing of Corrosion Thickness in Coated Steel Structures with THz-TDS. Measurement.

